# Shikonin induced Apoptosis Mediated by Endoplasmic Reticulum Stress in Colorectal Cancer Cells

**DOI:** 10.7150/jca.65297

**Published:** 2022-01-01

**Authors:** Hui Qi, Xing Zhang, Huanhuan Liu, Meng Han, Xuzhen Tang, Shulan Qu, Xiaoyu Wang, Yifu Yang

**Affiliations:** 1 Experiment Center for Science and Technology, Shanghai University of Traditional Chinese Medicine, Shanghai, 201203, P.R. China.; 2 Oncology and Immunology BU, Research Service Division, WuXi Apptec, Shanghai, China.

**Keywords:** Shikonin, Colorectal cancer, Endoplasmic reticulum stress, Apoptosis

## Abstract

Shikonin is a naphthoquinone pigment isolated from the root of Lithospermum erythrorhizon, which has displayed potent anti-tumor properties. However, the effects of shikonin in colorectal cancer cells have not been yet fully investigated. In this study, we demonstrated that shikonin significantly inhibited the activity of colorectal cancer cells in a time- and dose-dependent manner. The flow cytometry and western blot results indicated that shikonin induced cell apoptosis by down-regulating BCL-2 and activating caspase-3/9 and the cleavage of PARP. The expression of BiP and the PERK/elF2α/ATF4/CHOP and IRE1α /JNK signaling pathways were upregulated after shikonin treatment. The pre-treatment with N-acetyl cysteine significantly reduced the cytotoxicity of shikonin. Taken together, shikonin could inhibit proliferation of the colorectal cancer cell through the activation of ROS mediated-ER stress. The *in vivo* results showed that shikonin effectively inhibited tumor growth in the HCT-116 and HCT-15 xenograft models. In conclusion, shikonin inhibited the proliferation of colorectal cancer cells *in vitro* and *in vivo* and warrants future investigation.

## Introduction

Colorectal cancer (CRC) is one of the leading causes of cancer related deaths worldwide 1. According to the latest data published by the National Cancer Center of China, annually there are more than 420,000 individuals diagnosed with colorectal cancer, accounting for 9.88% of the newly diagnosed cancers in China 2.

Treatments which provide a limited increase of overall survival for colorectal cancer are surgical resection, radiotherapy and chemotherapy 3. Immunotherapy and targeted therapy with monoclonal antibodies can also improve the outcomes in some patients, but in many others the benefits are only modest or lacking 4, 5. Thus, there is an urgent need for new bioactive molecules with anti-cancer properties. In the traditional Chinese medicine, different natural products have been demonstrated to possess antitumor activities 6.

Nowadays, several natural products have been successfully used in cancer therapy, such as, paclitaxel (Taxol) that discovered in the bark of a Pacific yew tree, which has been commonly used in the treatment of breast, ovarian and other cancers 7. Shikonin is a naturally occurring naphthoquinone isolated from the root of the plant *Lithospermum erythrorhizon*, which has been widely used in the treatment of various disease 8. In the recent years, studies have shown that shikonin and its derivatives have antitumor effects in wide range of cancer types including breast 9, lung 10, ovarian 11, liver 12, prostate 13, gastric 14 and pancreatic cancer 15. However, the effects of shikonin in colorectal cancer cells have not been yet fully investigated yet.

Endoplasmic reticulum (ER) is a relatively complex organelle which regulates protein synthesis, folding, assembly and trafficking by utilizing its dynamic structural change 16. Therefore, it is necessary to ensure the homeostasis of endoplasmic reticulum environment. Alteration of ER stability will prime a series of signaling events known as endoplasmic reticulum stress (ERS). Although the main function of unfolded protein response is to restore the organelle's homeostasis, continuous ER stress and unfolded protein response can also cause cell death. One study published recently showed that shikonin induces apoptosis in human melanoma A375 cells through activating reactive oxygen species (ROS)-mediated endoplasmic reticulum stress and mitochondrial apoptotic pathway 17. Meanwhile, there are growing studies reveal that ROS and ER stress play an essential role in the development of colorectal cancer. For example, one study has shown that ER stress-related ATF6 upregulates CIP2A, which results in poor prognosis of colon cancer 18. ER stress induces apoptosis through inducing ROS, and that elevated ROS also play an important role in down-regulating cell cycle progression and cell survival. Therefore, targeting ER stress is a potential new option in CRC therapy. We hypothesized that shikonin have antitumor activity against human colorectal cancer, and tested this hypothesis in both *in vitro* and *in vivo* prospective.

## Materials and Methods

### Materials and reagents

The colorectal cancer cell lines HCT-116 (ATCC^®^ CCL-247™) and HCT-15 (ATCC^®^ CCL-225™) were purchased from the American Type Culture Collection (ATCC, USA). Shikonin was purchased from Meilun Biotechnology Co., Ltd (Shanghai, China). Trypsin was purchased from Gibco (New York, USA). Dimethyl sulfoxide (DMSO), tween 80, poly (ethylene glycol) 400 and N-acetyl cysteine (NAC) were obtained from Sigma (St. Louis, MO, USA).

### Cell culture and treatment

HCT-116 and HCT-15 cells were maintained in McCoy's 5A (Invitrogen, Carlsbad, USA) or RPMI-1640 medium containing 10% heat inactivated fetal bovine serum (Gibco, New York, USA) at 37 ºC in a 5% CO_2_ atmosphere. Cells were treated with different doses of shikonin at different time points during several experiments.

### Cell viability assay

Cell viability was examined by the CellTiter-Glo luminescent cell viability assay kit (Promega, Madison, WI, USA). Cells were seeded in 96-well plates at densities of 5000 cells (HCT-116) and 6000 cells (HCT-15) per well and treated with shikonin at various time points. After treatment, luminescence was recorded utilizing a Perkin Elmer 2104 EnVision plate reader (PerkinElmer, Hopkinton, MA).

### Cell colony formation

After treatment, cells were seeded in 6-well plates at a density of 1000 cells per well and cultivated for 10 days. Then, the cells were fixed with 4% paraformaldehyde (Sigma, St. Louis, MO, USA) for 30 min and stained with 1% crystal violet (Beyotime, Shanghai, China) for 1 h at room temperature. After that, the 6-well plates were washed with PBS. Images were taken using a microscope (Leica, Wetzlar, Germany) and the total number of cell colonies was counted.

### Apoptosis assay

Cells were seeded in 6-well plates at a density of 3 × 10^5^ cells per well and treated with shikonin. After 24 h treatment, the cells were harvested and stained with Annexin V-FITC and propidium iodide (PI) according to the manufacturer's instructions (BD Biosciences, San Diego, CA, USA). The cell apoptosis rate was detected by flow cytometry (FACSCanto II, BD Biosciences, USA).

### qRT-PCR

Total RNAs were extracted using the RNeasy mini kit (Qiagen, Hilden, Germany). The cDNA was synthesized using the cDNA reverse transcription kit (ABI, Carlsbad, USA). The qRT-PCR was performed using the Real-Time PCR System and the SYBR^®^ Green PCR Master Mix (ABI, Carlsbad, USA). QuantStudio7 (ABI, Carlsbad, USA) was used to acquire real-time quantitative PCR data. RNA expression was quantified using the 2^-ΔΔCt^ method. The sequences of the primer are shown in Table 1.

### Western blotting analysis

The total protein was extracted by using RIPA lysis buffer containing protease and phosphatase inhibitors (Sigma, St. Louis, MO, USA). The cell extracts were centrifuged at 15,000 rpm for 20 min at 4 °C. The protein concentrations were measured by using the pierce™ BCA protein assay kit (Thermo Scientific, Lithuania). A total of 20 μg of protein per sample was separated on SDS-PAGE gels and transferred onto PVDF membranes. After blocking with 5% nonfat dry milk, the PVDF membranes were incubated overnight with the primary antibody at 4 °C, then incubated for 2 h with the HRP-conjugated secondary antibody at room temperature. Later, the HRP substrate from West Femto Maximum Sensitivity kit (Thermo Scientific, Lithuania) was added to the PVDF membranes. Luminescence was detected with ImageQuant LAS 4000 (GE, USA).

### Xenograft mouse model

Female BALB/c nude mice were purchased from Shanghai Sino-British BK Laboratory Animal Co., LTD (Shanghai, China). Mice were injected with HCT-116 or HCT-15 tumor cells (5 × 10^6^) at the right flank. When the tumor volume reached up to approximately 100 mm^3^, mice were randomly assigned into two groups. Shikonin was injected intraperitoneally three times per week, for two weeks. After therapy, tumor sizes were measured twice weekly with calipers. The volume was expressed in mm3 using the formula: Tumor Volume = 0.5 a × b^2^, where a and b are the long and short diameters of the tumor, respectively. In another study, six mice were randomly grouped into two groups, after three doses, the tumor tissues were collected to conduct western blot. Mice were monitored daily for general health, activity, and well-being. During the study, the care and use of animals were conducted in accordance with the guidelines approved by the Institutional Animal Care and Use Committee (IACUC) of WuXi AppTec.

### Statistical analysis

All experiments were performed in biologically independent triplicates. Results are presented as mean ± standard deviation (SD). Comparisons between different treatment groups were tested using one-way analysis of variance (ANOVA). A value of p < 0.05 was considered to be statistically significant. Double asterisks (**) indicate p < 0.01, triple asterisks (***) indicate p < 0.001.

## Results

### Shikonin inhibited the proliferation of colorectal cancer cells

**Figure 1:**
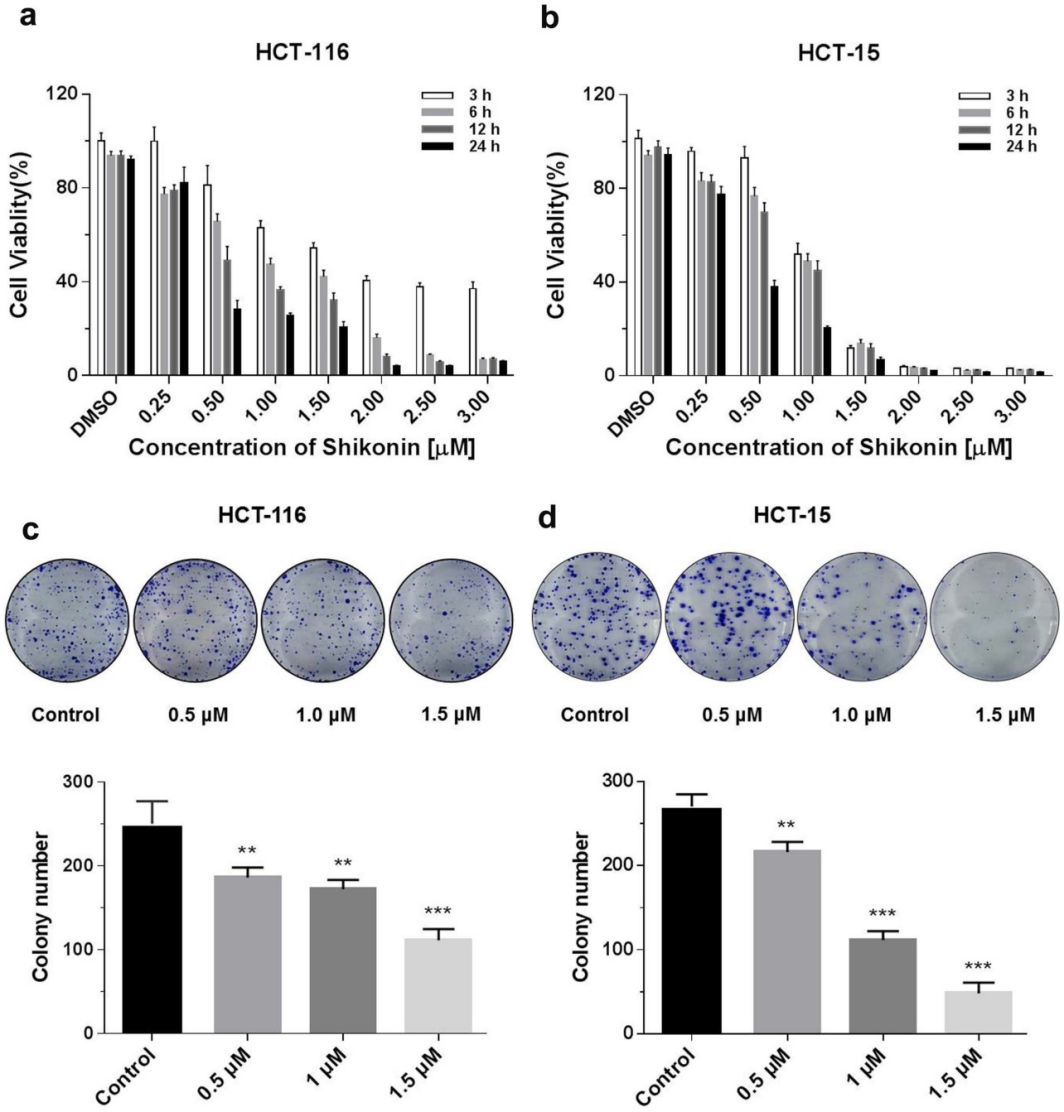
** Shikonin inhibited the proliferation of colorectal cancer cells**.** (a)** Shikonin inhibited colorectal cancer cell viability. HCT-116 and HCT-15 cells were treated with different doses (0.25, 0.5, 1, 1.5, 2, 2.5 and 3 µM) of shikonin at various time points (3, 6, 12 and 24 h). **(b)** Colony formation and number of colonies per well. DMSO was used as a negative control. Data are presented as mean ± SD of three independent experiments. Significance is indicated by * *p* < 0.05, ** *p* < 0.01 and *** *p* < 0.001 compared to the control group.

The cytotoxicity of shikonin on HCT-116 and HCT-15 colorectal cell lines was investigated using the Promega CellTiter-Glo luminescent cell viability assay kit. Colorectal cells were treated with different doses (0.25, 0.5, 1, 1.5, 2, 2.5 and 3 μM) of shikonin at various time points (3, 6, 12 and 24 h). Shikonin significantly inhibited the proliferation of colorectal cancer cells in a dose- and time-dependent manner (Figure 1a). The detailed statistic has been included in supplement 1 and supplement 2. HCT-15 cells were more sensitive to shikonin treatment compare with HCT-116. Moreover, cell colonies decreased at 10 days after treatment of shikonin (0.5 μM and higher doses), compared with the control group. Finally, a significant inhibition of colony formation was observed in the treatment of shikonin under the dosage of 1.5 μM (Figure 1b).

### Shikonin induced apoptosis of colorectal cancer cells

**Figure 2:**
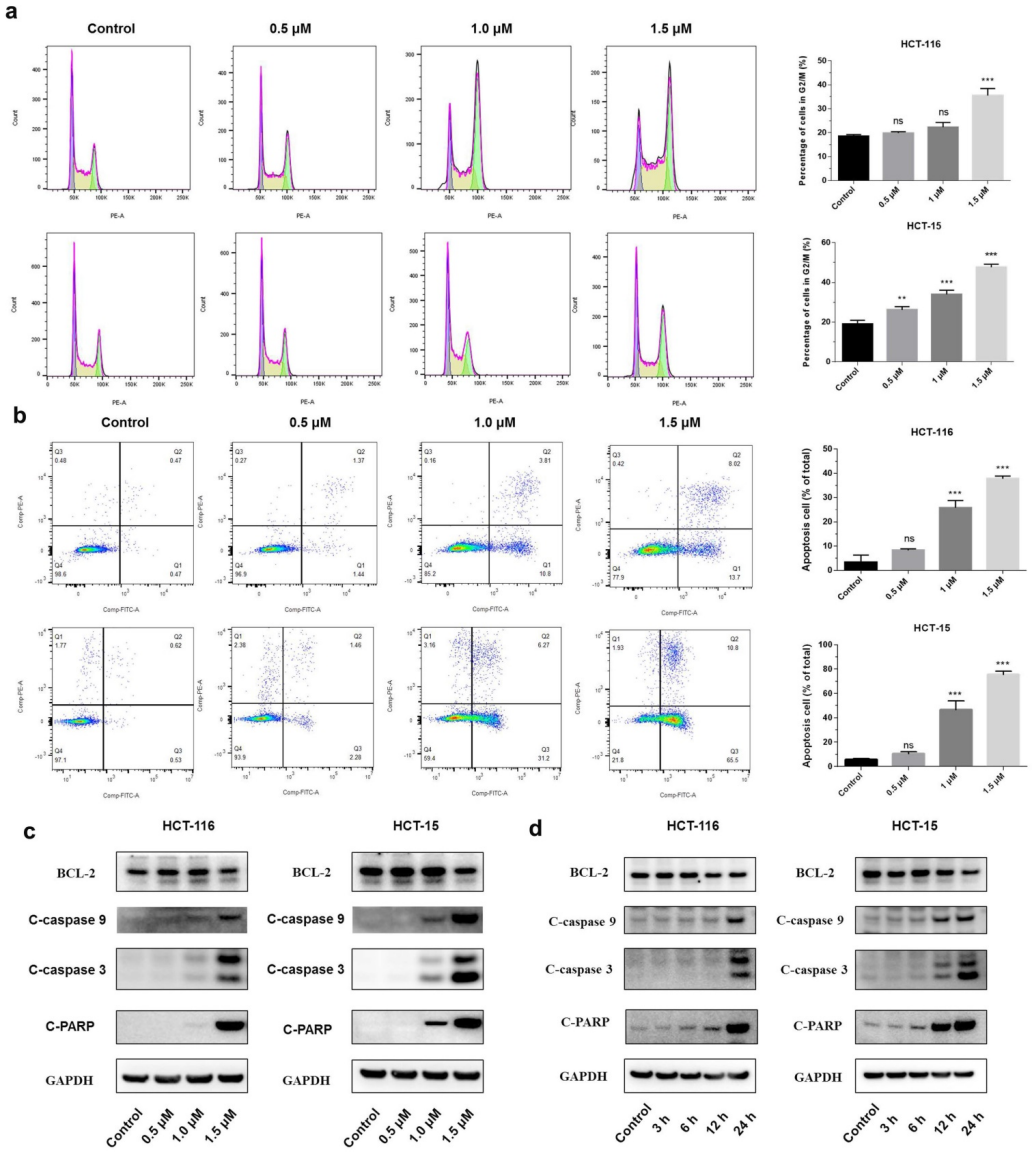
** Shikonin treatment induced apoptosis of colorectal cancer cells**.** (a-b)** Cell cycle and cellular apoptosis were determined by flow cytometric analysis. HCT-116 and HCT-15 cells were treated with shikonin for 24 h. **(c)** Western blot was performed to evaluate the apoptosis-related proteins after shikonin treatment. Cells were treated with different doses of shikonin (0, 0.5, 1, and 1.5 µM) for 24 h. **(d)** Western blot was performed to evaluate the apoptosis-related proteins after shikonin treatment. Cells were treated with shikonin (1.5 µM) at different time points (3, 6, 12, and 24 h). Data are presented as mean ± SD of three independent experiments. Significance is indicated by * *p* < 0.05, ** *p* < 0.01 and *** *p* < 0.001 compared to the control group.

A significant increase in the percentage of the G2/M phase was observed in both HCT-116 and HCT-15 cells after the treatment with shikonin (Figure 2a). The flow cytometry showed that shikonin induced apoptosis in a dose-dependent manner, with the percentages of cell apoptosis being 8.25%, 25.8%, 37.8%, and 10.62%, 46.80%, and 75.53% in HCT-116 and HCT-15 cells, respectively (Figure 2b). Western blot results showed that shikonin down regulated the expression level of anti-apoptotic Bcl-2, induced the cleavage of PARP and activated the caspase-3/9 (Figures 2c-d).

### Shikonin induced endoplasmic reticulum stress

**Figure 3:**
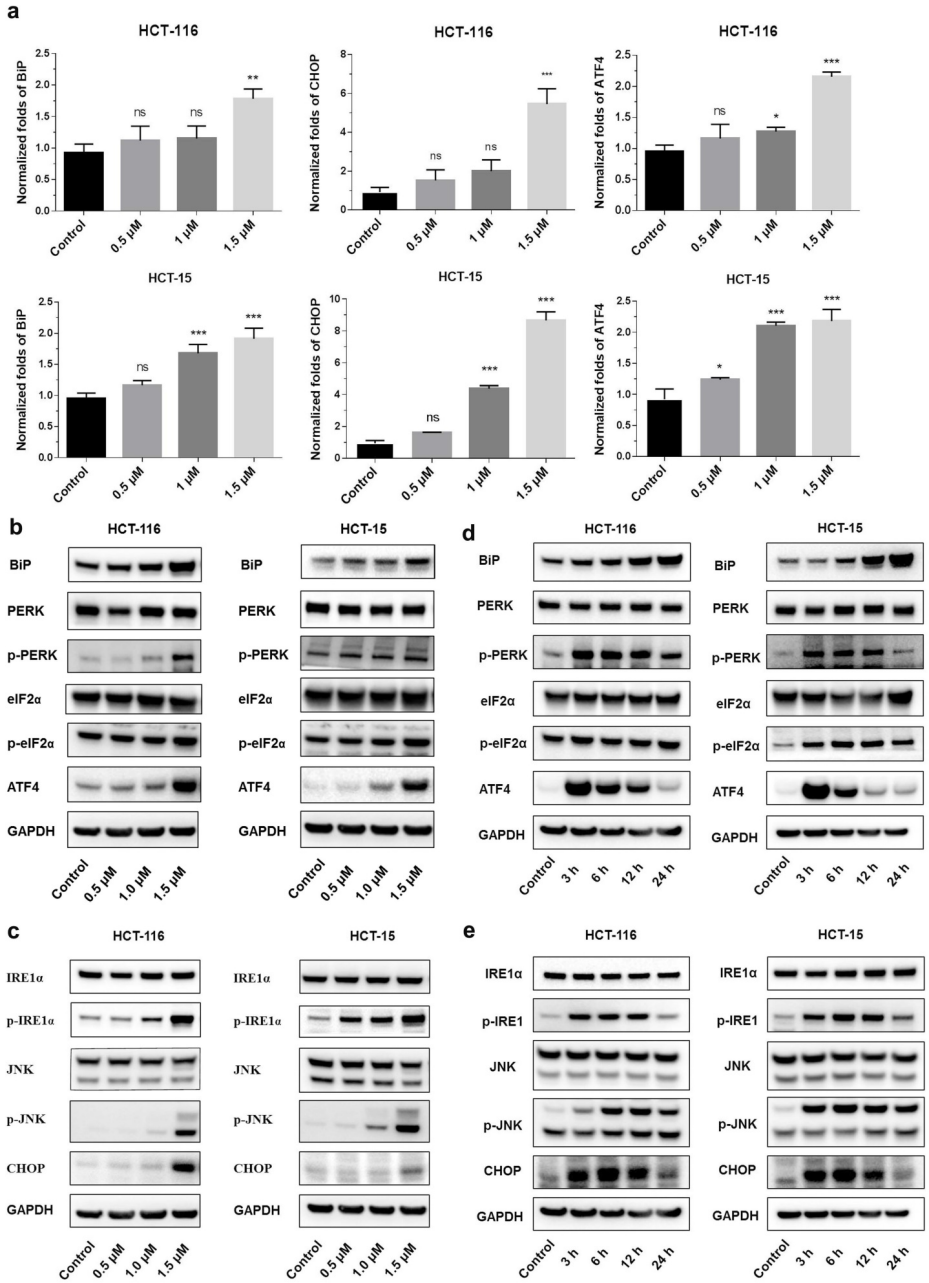
** Shikonin induced apoptosis of colorectal cancer cells. (a)** Gene expression levels of BiP, ATF4 and CHOP were analyzed by qRT-PCR. **(b-c)** Protein expression of PERK and IRE1α pathways after shikonin treatment. Cells were treated with different doses of shikonin (0, 0.5, 1, and 1.5 µM) for 24 h. **(d-e)** Protein expression of PERK and IRE1α pathways after shikonin treatment. Cells were treated with shikonin (1.5 µM) at different time points (0, 3, 6, 12, and 24 h). Data are presented as mean ± SD of three independent experiments. Significance is indicated by * *p* < 0.05, ** *p* < 0.01 and *** *p* < 0.001 compared to the control group.

Endoplasmic reticulum stress (ER stress) occurs when proteins are not properly folded or conformed. Multiple studies have described the relation between ER stress and cancer 19, 20. Consistent with this concept, we extracted RNA from shikonin treated HCT-116 and HCT15, examined the expression level of the principal genes involved in endoplasmic reticulum (ER) stress by conducting RT_PCR. Results showed that the expression of binding immunoglobulin protein (BiP), ATF4 and CHOP were significantly increased after shikonin treatment in HCT116 and HCT15 cells (Figures 3a-c). The phosphorylation pattern of protein kinase R-like ER kinase (P-ERK) pathway was also assessed. Western blot found that protein level of p-PERK, p-eIF2α and ATF4 increased in HCT116 and HCT15 cells after shikonin treatment (Figure 3b-c). Regarding the IRE1α pathway, shikonin treatment showed similar phosphorylation patterns in colorectal cancer cells. Shikonin significantly increased the protein level of p-IRE1α, p-JNK and CHOP in HCT116 and HCT15 cells (Figure 3d-e).

### Shikonin induced reactive oxygen species-mediated ER stress

**Figure 4:**
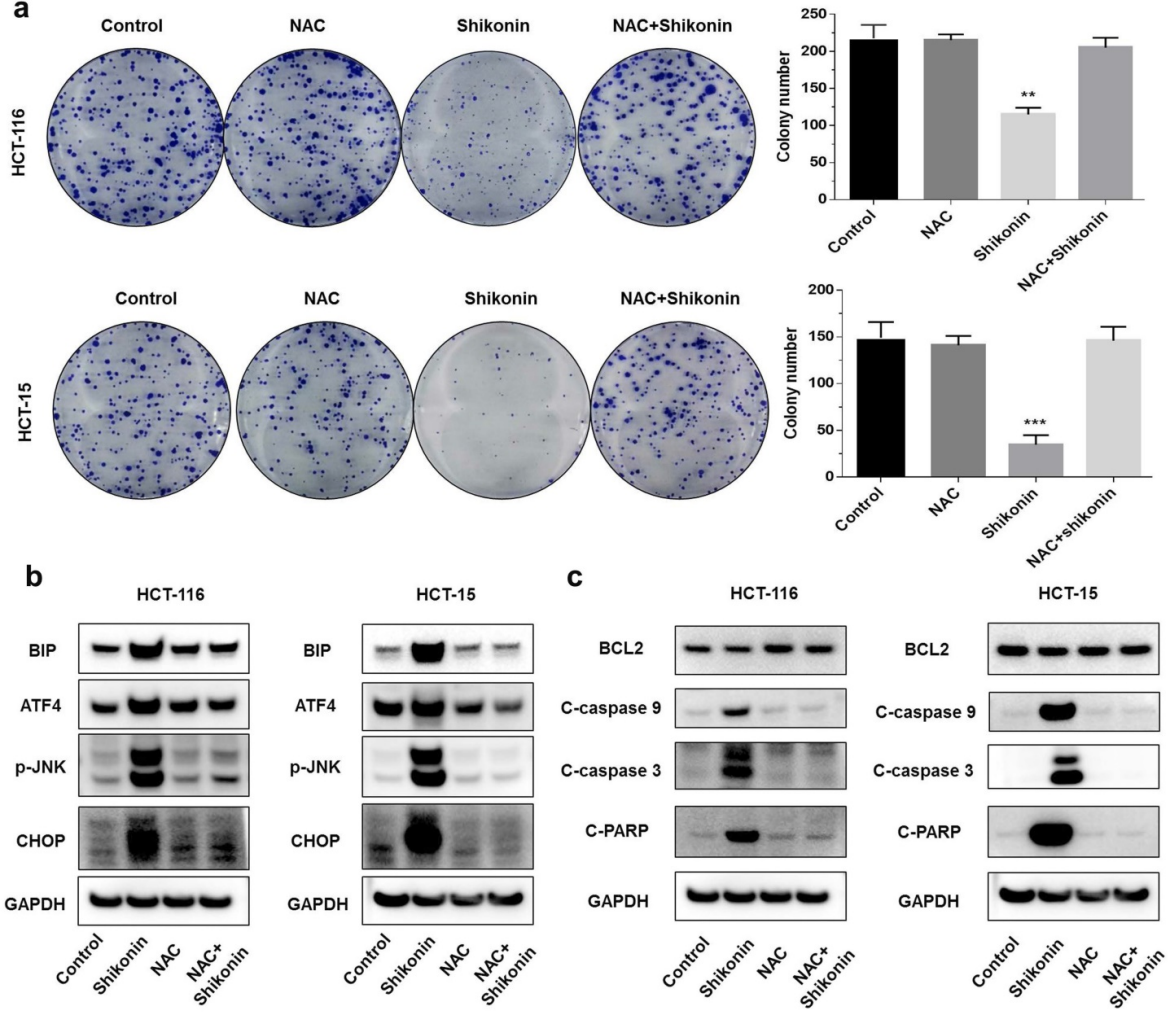
** Shikonin induced reactive oxygen species-mediated ER stress**.** (a)** Colony formation and number of colonies per well. Cells were pre-treated with N-acetyl cysteine (NAC) for 2 h and then treated with shikonin (1.5 µM) for 10 days. **(b-c)** Western blot evaluated the apoptosis-related proteins and ER stress-related proteins when cells were pre-treated with NAC for 2 h and then treated with shikonin (1.5 µM) for 24 h. DMSO was used as a negative control. Data are presented as mean ± SD of three independent experiments. Significance is indicated by * *p* < 0.05, ** *p* < 0.01 and *** *p* < 0.001 compared to the control group.

Shikonin is an inducer of reactive oxygen species 21, 22. A previous study found that shikonin induced apoptosis through the production of reactive oxygen species. To investigate the pro-apoptotic mechanisms of shikonin, we pre-treated the colorectal cancer cells with N-acetyl cysteine (NAC) for 2 hours before treating with shikonin. The colony formation assay showed that the pre-treatment with NAC reduced the cytotoxic effects of shikonin significantly (Figure 4a). Pre-treatment with NAC also significantly suppressed the shikonin-induced expression of BiP, p-JNK, ATF4 and CHOP (Figure 4b). The protein level of cleaved- caspase-3/9 and cleaved-PARP were completely neutralized in the presence of NAC (Figure 4c).

### Shikonin suppressed the growth of colorectal cancer cells *in vivo*

**Figure 5:**
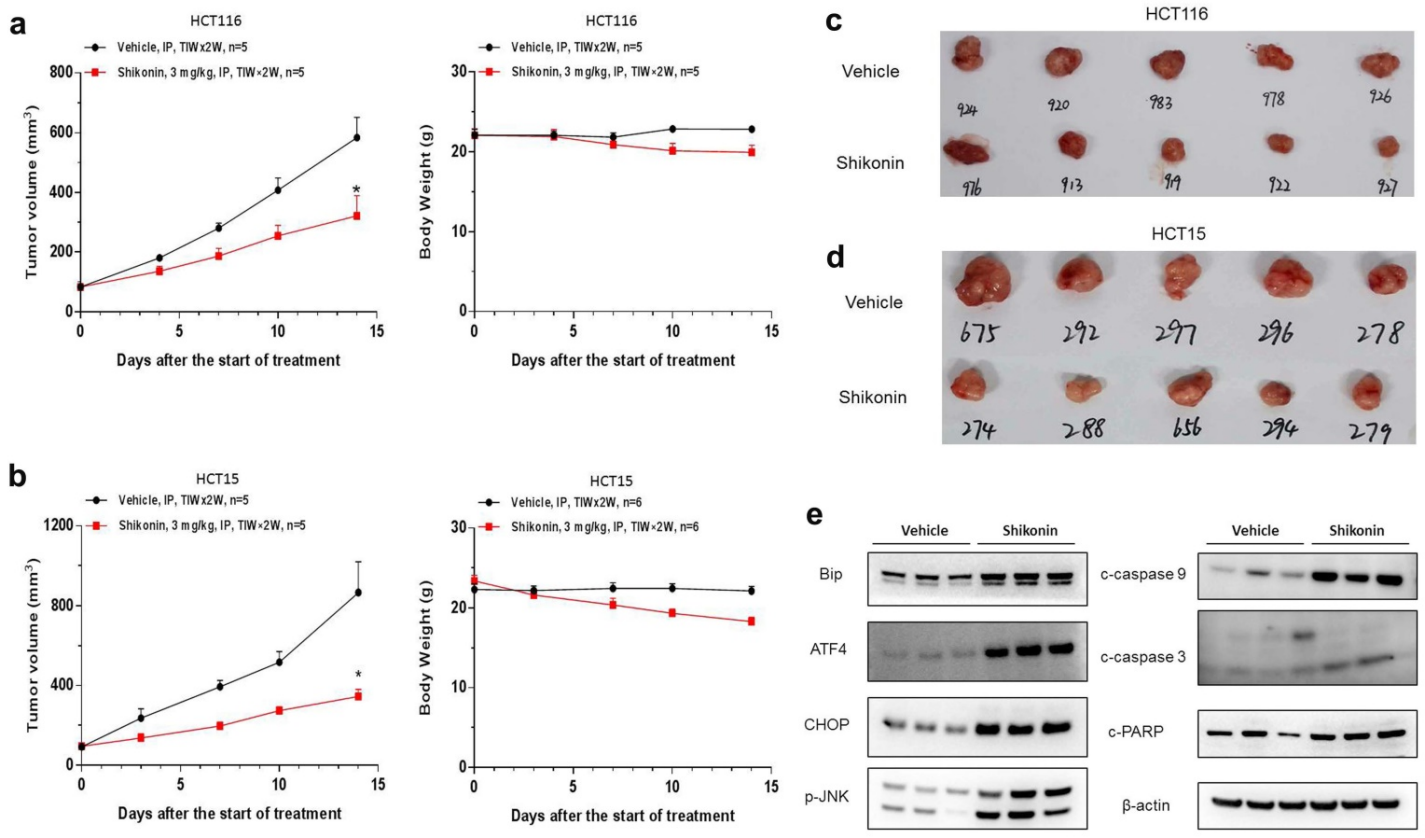
** Shikonin suppressed the growth of colorectal cancer cells *in vivo***. HCT-116 and HCT-15 cells were implanted subcutaneously in BALB/c nude mice. The tumor-bearing mice were administered with shikonin (3 mg/kg) or vehicle via intraperitoneal injection for two weeks (n = 5 per group). **(a-b)** The tumor growth curve and the body weight change of HCT-116 and HCT-15 cells treated with shikonin. **(c-d)** The representative images of tumors from the implanted mice with HCT-116 and HCT-15 cells.** (e)** Western blot was performed to evaluate the protein level after shikonin treatment in HCT-15 xenograft model. Data are shown as mean ± SD. Significance is indicated by * *p* < 0.05 compared to the control group.

To investigate should shikonin have anticancer activity *in vivo*, HCT-116 and HCT-15 cells were implanted subcutaneously in BALB/c nude mice. Shikonin effectively inhibited tumor growth *in vivo*, with the tumor growth inhibition (TGI) rates of HCT-116 and HCT-15 cells being 52.3% and 67.8%, respectively (Figure 5a-d). Please note that this is the primary study, which means the dosing schedule and roots could be optimized to archive better efficacy. Furthermore, western blot was conducted to evaluate the protein levels in HCT-15 tumor tissues collected from anther *in vivo* study. We also found that the expression of BiP, ATF4, CHOP and p-JNK were significantly increased after shokonin treatment. Moreover, cleaved caspase-3/9 and cleaved PARP were also upregulated in the tumor tissues (Figure 5e). Taken together, these results showed that shikonin has a powerful antitumor effect *in vivo*.

## Discussion

Shikonin is a major naphthoquinone compound found in the roots of *Lithospermum erythrorhizon* that exhibits powerful anticancer activities. Shikonin also exerts other activities, including antimicrobial, anti-inflammatory, and antioxidant 23, 24. Most of the studies on shikonin were focused on cellular responses, while the effects of shikonin *in vivo* are largely unknown 25, 26. In the current study, we found that shikonin induced apoptosis in colorectal cancer cells through activating ER stress and upregulate reactive oxygen species, antioxidants treatment could neutralize the induction *in vitro*.

Previous studies found that shikonin induce cell cycle arrest in G0/G1 phase and G2/M phase, thereby inhibiting cancer cell proliferation 27, 28. In accordance, we demonstrated that shikonin induced G2/M phase arrest, down-regulated protein level of Bcl-2, along with increased protein level of cleaved-PARP and cleaved-caspase-3/9 suggesting that apoptosis was elevated in both HCT-116 and HCT-15 cells. Study has reported that Shikonin-induced apoptosis through the mitochondrial pathway, and generation of reactive oxygen species, the activation of c-Jun-N-terminal kinase (JNK), p38, caspase-3/9 and the cleavage of PARP 29.

Cancer cells grow continuously, increase reactive oxygen species and induce hypoxia, thus activating ER stress. ER stress in turn activates the unfolded protein response (UPR), that is both apoptotic and adaptive in tumor cells. The purpose of the UPR is to balance the ER folding environment under stress. If the stress is prolonged and the UPR fails to restore ER homeostasis, tumor cells will undergo apoptosis. It has been reported that shikonin induced ER stress-mediated apoptosis in melanoma cells via the production of reactive oxygen species and the up-regulation of p-eIF2α, CHOP and caspase-3 17. In colon cancer cells, the main components of shikonin-induced apoptosis were the generation of reactive oxygen species, the down-regulation of Bcl-2 and the activation of the caspase cascade 30. In the present study, we found that shikonin induced the cell apoptosis of colorectal cancer cells by activating the ER stress through the PERK/elF2α/ATF4/CHOP and IRE1α/JNK signaling pathways, up-regulating the anti-apoptotic protein Bcl-2 and increasing the expression of caspase-3/9. There is a functional crosstalk between the production of reactive oxygen species, the mitochondrial apoptosis pathway and the ER-stress apoptosis pathway, which leads to cell death in colorectal cancer cells.

In our previous *in vivo* studies, oral administration of shikonin did not show antitumor activities in colorectal cancer xenograft models (supplement 3). Here, we found that shikonin effectively inhibited tumor growth through intraperitoneal injection (3 mg/kg, 3 times per week). It is possible that the anti-tumor activities of shikonin are more evident when injected intraperitoneally, however, PK analyze is required to compare differences of* in vivo* exposures and metabolites between oral and intraperitoneal injection. The new treatment schedule and new option of dosing roots should have great potential to improve efficacy of shikonin in xenograft mouse models.

ER stress and mitochondrial apoptotic pathway contribute to tumorigenesis in wide range of cancer types 31. Shikonin can activate the apoptosis-related signal regulation network and reduce the expression of Bcl-2 by interfering the PI3K/AKT signaling pathway in afatinib-resistant non-small cell lung cancer cell lines H1650/R and H1975/R *in-vitro* 32. However, these studies didn't conduct studies in xenograft models. Our study found that intraperitoneal injection gave better efficacy comparing with oral administration, therefore, intraperitoneal injection of shikonin should have potential to give promising efficacy against afatinib-resistant non-small cell lung cancer. Taken together, shikonin should have great potential in treatments of variety cancer types, and more studies need to be conducted in investigate the mechanism of action and druggability.

## Supplementary Material

Supplementary figure and tables.

## Figures and Tables

**Table 1:** The primers used for quantitative PCR

Gene	Primer sequence (5'---3')	Base (bp)
BiP forward	CACAGTGGTGCCTACCAAGA	20
BiP reverse	TGTCTTTTGTCAGGGGTCTTT	21
ATF4 forward	CCCTTCACCTTCTTACAACCTC	22
ATF4 reverse	TGCCCAGCTCTAAACTAAAGGA	22
CHOP forward	AGCCAAAATCAGAGCTGGAA	20
CHOP reverse	TGGATCAGTCTGGAAAAGCA	20
Bcl-2 forward	GGTCATGTGTGTGGAGAGAGCG	22
Bcl-2 reverse	CCGTACAGTTCCACAAAGGC	20
GAPDH forward	GGAGTCAACGGATTTGGTCG	21
GAPDH reverse	ACGGTGCCATGGAATTTGC	19
