# Supplementary material for: Shikonin induced Apoptosis Mediated by Endoplasmic Reticulum Stress in Colorectal Cancer Cells
Source: J Cancer. 2022 Jan 1;13(1):243–52. doi: 10.7150/jca.65297 (PMC8692675; doi:10.7150/jca.65297)

Supplement 1. Cell viability of shikonin on HCT-116 cells

	Shikonin treatment (Mean ± SD)			
**Concentration (μM)**	**3 h**	**6 h**	**12 h**	**24 h**
Control	100.13 ± 3.30	93.88 ± 1.74	93.92 ± 1.84	92.24 ± 1.43
0.25	99.9 ± 6.07	77.39 ± 2.85***	79.00 ± 2.33***	82.24 ± 6.64 ***
0.5	81.28 ± 8.27***	65.74 ± 3.22***	49.29 ± 5.80***	28.32 ± 3.75***
1	63.15 ± 2.91***	47.33 ± 2.68***	36.66 ± 1.22***	25.73 ± 0.95***
1.5	54.34 ± 2.29***	42.22 ± 2.75***	32.17 ± 3.05***	20.68 ± 2.26***
2	40.51 ± 2.02***	16.11 ± 1.51***	8.26 ± 0.88***	4.01 ± 0.43***
2.5	37.7 ± 1.87***	8.73 ± 0.51***	5.95 ± 0.39***	4.26 ± 0.20***
3	37.01 ± 2.90***	6.97 ± 0.50***	7.06 ± 0.61***	6.16 ± 0.31***

Supplement 2. Cell viability of shikonin on HCT-15 cells

	Shikonin treatment (Mean ± SD)			
**Concentration (μM)**	**3 h**	**6 h**	**12 h**	**24 h**
Control	101.49±3.31	94.04±2.10	97.87±2.52	94.57±2.68
0.25	95.82±1.69*	83.20±3.57***	82.67±3.12***	77.56±3.24***
0.5	93.20±4.77***	76.72±3.76***	69.93±3.88***	37.90±2.83***
1	51.94±4.63***	48.96±3.17***	45.09±4.05***	20.53±0.70***
1.5	11.72±1.13***	13.76±1.66***	11.71±2.01***	6.80±1.14***
2	3.90±0.57***	3.44±0.51***	3.21±0.16***	2.12±0.11***
2.5	3.21±0.18***	2.35±0.23***	2.46±0.13***	1.64±0.09***
3	3.23±0.13***	2.50±0.17***	2.64±0.09***	1.68±0.10***

Supplement 3. ***In vivo* efficacy studies of shikonin in colorectal cancer xenograft models**. HCT-116 and HCT-15 cells were implanted subcutaneously in BALB/c nude mice. The tumor-bearing mice were oral administered with shikonin (20 mg/kg) or vehicle for two or three weeks. **(a)** The tumor growth curve and the body weight change in HCT-116 xenograft model. **(b)** The tumor growth curve and the body weight change in HCT-15 xenograft model.

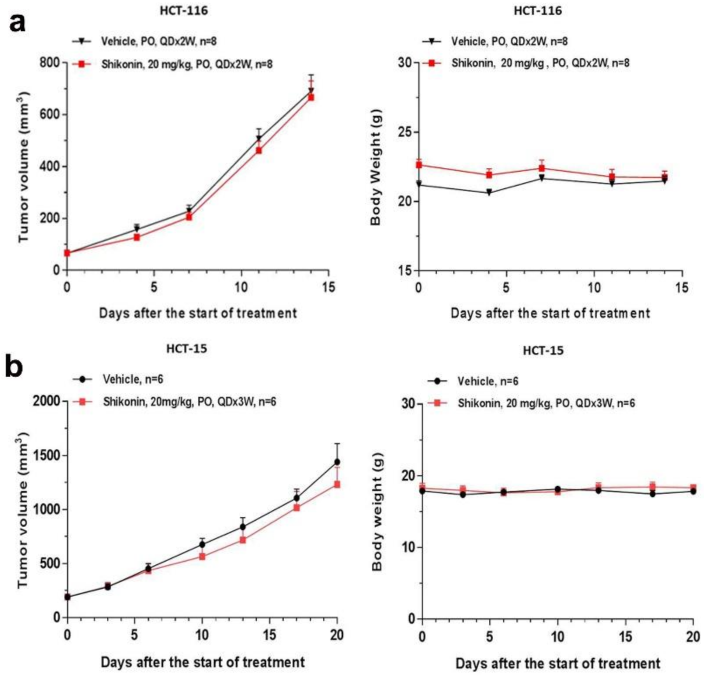

Supplement: Supplementary file 1 — Supplementary figure and tables. [file jcav13p0243s1.pdf]
